# Efficacy and safety of low- to medium-dose telitacicept in adults with high-risk progressive IgA nephropathy: a retrospective real-world study

**DOI:** 10.1186/s12882-025-04708-w

**Published:** 2025-12-17

**Authors:** Juan Pei, Xiuhua Xu, Ruihong Huang, Xuejuan Sun, Jun Zhang, Xing Chen, Lijing Yao, Hengyuan Zhang, Leping Shao, Yinan Li

**Affiliations:** 1 The School of Clinical Medicine, Fujian Medical University, Fuzhou, China; 2 Department of Nephrology, The First Affiliated Hospital of Xiamen University, School of Medicine, Xiamen University, Xiamen, China

**Keywords:** Telitacicept, IgA nephropathy, 24-hour urinary protein, Glomerular filtration rate, Urinary red blood cell count

## Abstract

**Objective:**

The purpose of this study was to evaluate the efficacy and safety of low- to medium-dose (160 mg/W or 80 mg/W) telitacicept in adults at high risk of progression IgA nephropathy.

**Methods:**

This was a single-center retrospective study. The study included adults with high risk progression of IgA nephropathy who were treated with telitacicept between November 2022 and April 2024 and followed for at least 24 weeks.

**Results:**

In this study of 11 patients, telitacicept significantly reduced proteinuria by 64.38% (*p* < 0.001) and increased eGFR by 23.18% (*p* = 0.005) at week 24. Both the 80 mg and 160 mg dose groups demonstrated significant reductions in proteinuria (68.67% and 59.23%, respectively, both *p* < 0.001) and urinary RBC counts. The overall response rate was 90.91%. Telitacicept demonstrated a favorable safety profile, with only mild injection-site reactions reported (18.2%) and no serious adverse events.

**Conclusion:**

In this real-world study, low- to medium-dose of telitacicept combined with conventional therapy showed rapid onset efficacy and safety for treating high-risk progressive IgA patients.

## Introduction

IgA nephropathy (IgAN), the most common primary glomerular disease in China, accounts for 24.09–35.80% of biopsy-diagnosed renal diseases and progresses to end-stage renal disease (ESRD) in 20–40% of patients within 10–20 years of initial clinical presentation [1, 2]. The disease exhibits heterogeneous clinical and pathological manifestations. Risk stratification in IgAN typically integrates clinical parameters such as persistent proteinuria, reduced estimated glomerular filtration rate (eGFR), hypertension, and histopathological findings from renal biopsy to identify patients at high risk of disease progression [3, 4].

The complex pathogenesis of IgAN is rooted in the “four-hit” hypothesis, in which B lymphocyte activation plays a crucial role [5]. B-lymphocyte stimulator (BLyS) and a proliferation-inducing ligand (APRIL) are critical cytokines that promote B cell survival, differentiation, and the production of antibodies, including Gd-IgA1 [6–8]. Telitacicept, a dual BLyS/APRIL inhibitor, demonstrated promising efficacy and safety in Phase II trials for IgAN [9, 10], with ongoing Phase III trials focusing on 160 mg and 240 mg doses. However, in the real world, the clinical value of telitacicept is still unclear. This study evaluates the efficacy and safety of low- to medium-dose of telitacicept (80–160 mg/week) in adults with high-risk progressive IgAN in real world.

## Patients and methods

### Study population

This single-center retrospective study included 11 adults with high-risk IgAN treated with telitacicept (160 mg/week or 80 mg/week) between November 2022 and April 2024, with ≥ 24 weeks of follow-up. Patients included met the following criteria [10]: (1) Biopsy-confirmed IgAN with high progression risk, defined as persistent proteinuria (> 0.5 g/day or urine protein-to-creatinine ratio > 0.5 mg/mg) despite ≥ 6 months of renin-angiotensin system inhibitors (RASI), steroids, or immunosuppressants, or significant steroid-related adverse effects [11, 12]; (2) Age ≥ 18 years; (3) eGFR ≥ 35 mL/min/1.73 m² (calculated using CKD-EPI formula). Patients were excluded from the study for the following reasons: Secondary IgAN, active infections, or malignancies. The study was conducted in accordance with the Declaration of Helsinki and was approved by the Ethics Committee of The First Affiliated Hospital of Xiamen University ((IRB approval No. 186 [2025]​), informed consent was obtained from all patients before receiving telitacicept treatment. Clinical trial number: not applicable.

### Data collection

This study was a single-center retrospective study. General patient information (gender, age, BMI, blood pressure) and renal pathology were collected. The last assessment results before treatment with different doses of Telitacicept (160 mg/W or 80 mg/W) were used and compared with the indicators at 12 weeks and 24 weeks of medication. The main observation indicators were urine protein quantification, plasma albumin (ALB), serum creatinine (CRE), eGFR, and urine red blood cell count. Secondary observation indicators were blood pressure, blood urea (BUN), hemoglobin (HB), white blood cell count, lymphocyte count, blood uric acid (UA), blood glucose (GLU), alanine aminotransferase (ALT), aspartate aminotransferase (AST), total cholesterol (CHO), and triglycerides (TG).

### Efficacy evaluation

(1) Complete remission (CR), urine protein quantification ≤ 0.3 g/24 h, and stable renal function (eGFR decrease ≤ 30%); (2) Partial remission (PR), urine protein quantification reduction ≥ 50%, and stable renal function, but not reaching complete remission; (3) Ineffective, not meeting the criteria of complete remission and partial remission.

### Outcomes

The Primary Outcomes included: Changes in 24-hour urinary protein, eGFR. Secondary outcomes included: Hematuria, adverse events, the rates of complete remission (CR), partial remission (PR) and no remission.

### Subgroup analyses

To explore potential differential treatment effects, pre-specified subgroup analyses were performed to compare the above outcomes (changes in proteinuria, eGFR, and hematuria at week 24) between the 80 mg and 160 mg dose groups, as well as between patients receiving telitacicept with versus without concomitant immunosuppression.

### Safety assessment

The occurrence of adverse events (AEs) and serious adverse events (SAEs) were carefully assessed. SAEs were classified according to predefined criteria: events posing life-threatening risks, resulting in mortality, necessitating unplanned hospitalization or prolongation of existing hospitalization, or inducing persistent/permanent organ dysfunction. The evaluation included injection site reactions, systemic or localized infections, cardio-cerebrovascular incidents, hepatotoxicity, gastrointestinal system damage, hematological system damage, reproductive system damage, osteonecrosis or pathological fractures and other clinically significant AEs.

### Statistical analysis

Data were analyzed using SPSS 19.0. For continuous variables, normally distributed data were presented as mean ± standard deviation (SD) and non-normally distributed data were presented as median [interquartile range (IQR) 25–75%]. Parametric tests such as a paired samples t test and non-parametric tests such as Wilcoxon rank sum tests were used to compare the differences at different time points (baseline, 12 weeks, and 24 weeks). All tests were two-sided, and a p-value of < 0.05 was considered statistically significant.

## Results

### Study population and baseline characteristics

The cohort comprised 11 patients (81.82% female; median age 35 years [interquartile range (IQR) 32–39]; The median time from renal biopsy to initiation of telitacicept was 13 months [IQR 7–23]), and the median duration of previous exposure to immunosuppressants was 12 months [IQR 6–22]. Baseline clinical parameters included mean 24-hour urinary protein excretion of 1.65 ± 0.78 g/24 h, mean estimated glomerular filtration rate (eGFR) of 67.59 ± 29.23 mL/min/1.73 m², and mean serum albumin of 40.09 ± 4.67 g/L. Concomitant immunosuppressive therapy was administered to 4 patients (36.4%): glucocorticoids alone (*n* = 2, distributed across dose groups), glucocorticoids plus mycophenolate mofetil (80 mg group, *n* = 1), and glucocorticoids plus tacrolimus (160 mg group, *n* = 1). There were no significant differences in baseline characteristics were observed between the telitacicept 80 mg and 160 mg groups. While concomitant medications remained stable throughout the treatment, glucocorticoid dosages were gradually reduced. The baseline characteristics of all patients are shown in Table 1.

**Table 1:** Baseline characteristics of study participants

Characteristics	Telitacicept 80 mg (*n* = 6)	Telitacicept 160 mg (*n* = 5)	All (*n* = 11)	*P*
Age, median (IQR), year	32 (28, 37)	38 (35,39)	35 (32, 39)	0.738
Female sex, n (%)	5 (83.33)	4 (80)	9 (81.82)	0.887
Weight, mean (SD), kg	56.17 ± 4.72	63.60 ± 4.64	59.55 ± 3.37	0.296
BMI, mean (SD), kg/m^2^	22.68 ± 1.58	24.45 ± 1.56	23.48 ± 1.09	0.450
Blood pressure, mean (SD), mmHg				
Systolic	116.33 ± 2.39	126.20 ± 3.77	120.82 ± 2.56	0.625
Diastolic	74.16 ± 1.19	78.00 ± 1.64	75.91 ± 1.12	0.963
Past systemic corticosteroid therapy, n (%)	6 (100)	5 (100)	11 (100)	-
Past other immunosuppressant therapy, n (%)	2 (33.33)	4 (80)	6 (54.55)	0.524
Concomitant medications with telitacicept, *n* (%)				
ACEI/ARBs	6 (100)	5 (100)	11 (100)	NA
SGLT2	0 (0)	3 (60%)	3 (27.27)	0.061
Prednisone only	1 (16.7)	1 (20)	2 (18.18)	1.000
Prednisone + Mycophenolate mofetil	1 (16.7)	0 (0)	1 (9.09)	0.455
Prednisone + Tacrolimus	0 (0)	1 (20)	1 (9.09)	1.000
Oxford classification, *n*				
M 0/1	0/6	0/5	0/11	NA
E 0/1	4/2	5/0	9/2	0.455
S 0/1	0/6	0/5	0/11	NA
T 0/1/2	4/2/0	2/2/1	6/4/1	0.452
C 0/1/2	1/5/0	2/3/0	3/8/0	0.545
Time since diagnosis before telitacicept treatment, median (IQR), months	23 (13,32)	7 (7,10)	13 (7,23)	0.051
Hemoglobin, mean (SD), g/L	113.67 ± 20.31	137.60 ± 16.68	124.54 ± 21.77	0.433
ALB, mean (SD), g/L	39.58 ± 5.95	40.70 ± 3.10	40.09 ± 4.67	0.414
Total triglyceride, median (IQR), mmol/L	0.96 (0.75, 1.26)	2.62 (1.00, 3.03)	1.10 (0.84, 2.83)	0.340
Total cholesterol, mean (SD), mmol/L	4.97 ± 0.45	6.46 ± 0.44	5.65 ± 0.38	0.872
Urea, mean (SD), mmol/L	7.82 ± 2.92	5.24 ± 2.86	6.64 ± 3.06	0.850
Creatinine, mean (SD), µmol/L	123.50 ± 53.63	94.00 ± 47.84	110.01 ± 50.90	0.737
Uric Acid, mean (SD), µmol/L	383.67 ± 109.04	355.00 ± 175.22	370.64 ± 135.83	0.651
eGFR, mean (SD), mL/min/1.73 m^2^	54.82 ± 16.96	82.92 ± 35.19	67.59 ± 29.23	0.662
24-hour proteinuria, mean (SD), g/day	1.25 ± 0.78	2.14 ± 0.50	1.65 ± 0.78	0.919
Urinary RBC count (cells/µl), median (IQR)	424.55 (70.10, 1403.60)	87.20 (54.90, 372.95)	121.60 (62.00, 641.30)	0.414

ACEI, angiotensin-converting enzyme inhibitor; ALB, albumin; ARBs, angiotensin receptor blockers; BMI, body mass index; CR, complete remission; eGFR, estimated glomerular filtration rate; IQR, interquartile range; NA, Not Available; PR, partial remission; RBC, red blood cell; SD, standard deviation; SGLT2, sodium-glucose cotransporter-2

### Primary outcomes

#### Effects on proteinuria

**Fig. 1:**
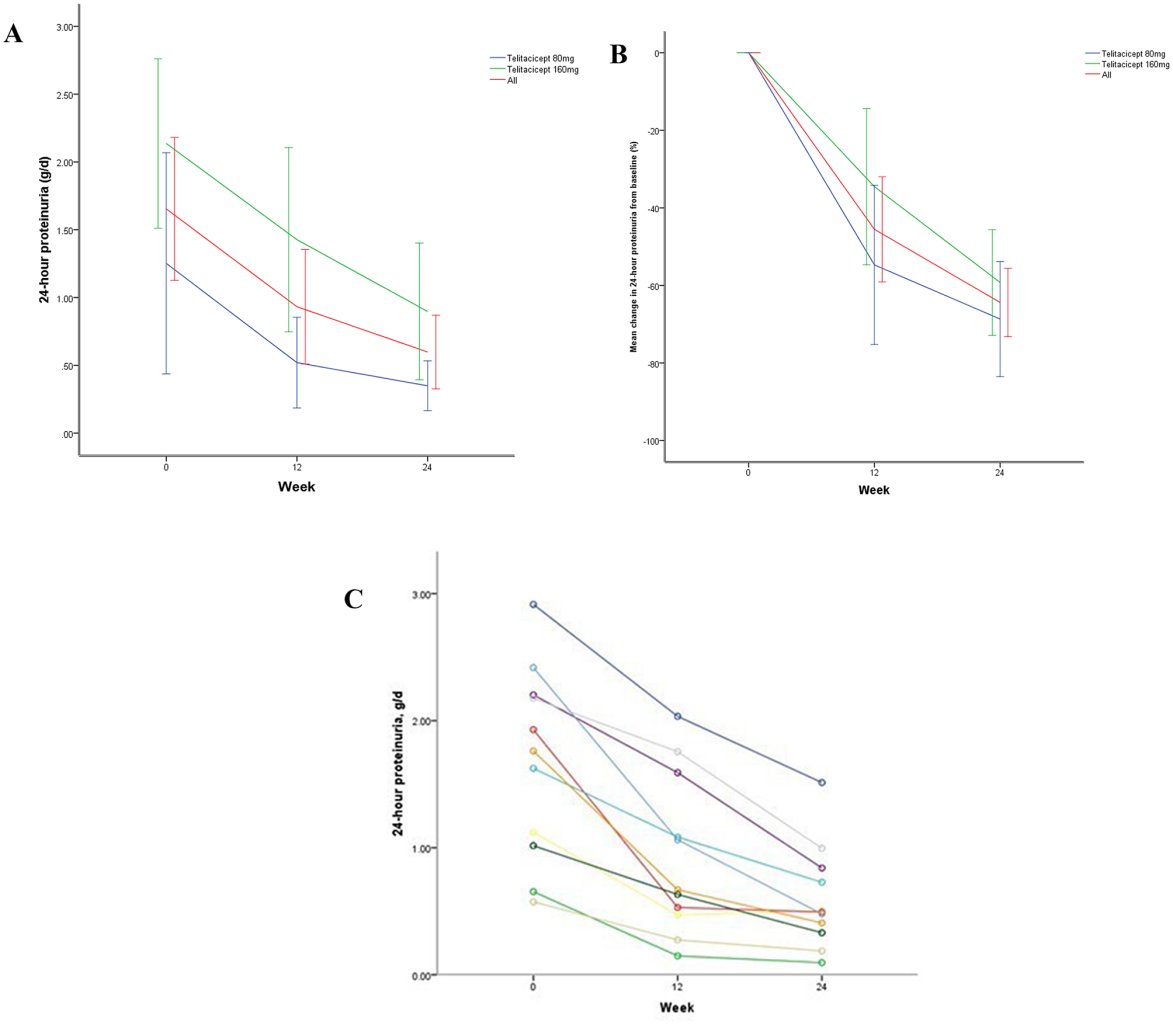
Changes in proteinuria at baseline, 12 weeks and 24weeks. (**a**) Absolute mean changes in 24-hour proteinuria from baseline in patients receiving telitacicept (80 mg/week or 160 mg/week). Data are expressed as mean values (bars indicate the standard error of the mean). (**b**) Mean change (%) in 24-hour proteinuria from baseline in patients receiving telitacicept (80 mg/week or 160 mg/week). (**c**) Individual trajectories of 24-hour urinary protein for each patient in the cohort

Significant proteinuria reduction was observed 12 weeks after initiating telitacicept, from 1.65 ± 0.78 g/24 h to 0.93 ± 0.62 g/24 h for 24-hour proteinuria, with mean reduction of 45.53% (95% CI: 31.97**–**59.09, *p* < 0.001). By week 24, the level of 24-hour proteinuria reduced to 0.60 ± 0.40 g/24 h, representing a reduction of 64.38% (95% CI: 55.56**–**73.21, *p* < 0.001). Similar to the trend observed in the full cohort, the level of 24-hour proteinuria in telitacicept 80 mg group at week 12 decreased from 1.25 ± 0.78 g/24 h to 0.52 ± 0.32 g/24 h, with mean decrease of 54.70% (95% CI: 34.19**–**75.72, *p* = 0.001)), to 0.35 ± 0.18 g/24 h at week 24, with mean decrease of 68.67% (95% CI: 53.83**–**85.51, *p* < 0.001). In telitacicept 160 mg group, the level of 24-hour proteinuria at week 12 decreased from 2.14 ± 0.50 g/24 h to 1.43 ± 0.55 g/24 h, with mean decrease of 34.53% (95% CI: 14.37**–**54.68, *p* = 0.009), to 0.90 ± 0.41 g/24 h at week 24, with mean decrease of 59.24% (95% CI: 45.61**–**72.88, *p* < 0.001). (Figs. 1a, b). The individual trajectories of proteinuria for all patients are provided in Figs. 1c.

A sensitivity analysis was performed after excluding the two patients who received concomitant tacrolimus or mycophenolate mofetil. In the remaining nine patients, significant proteinuria reduction was observed 24 weeks after initiating telitacicept, from 1.56 ± 0.83 g/24 h to 0.63 ± 0.44 g/24 h, with a mean reduction of 61.24% (95% CI: 51.71 to 70.78, *p* < 0.001).

#### Effects on estimated glomerular filtration rate

**Fig. 2:**
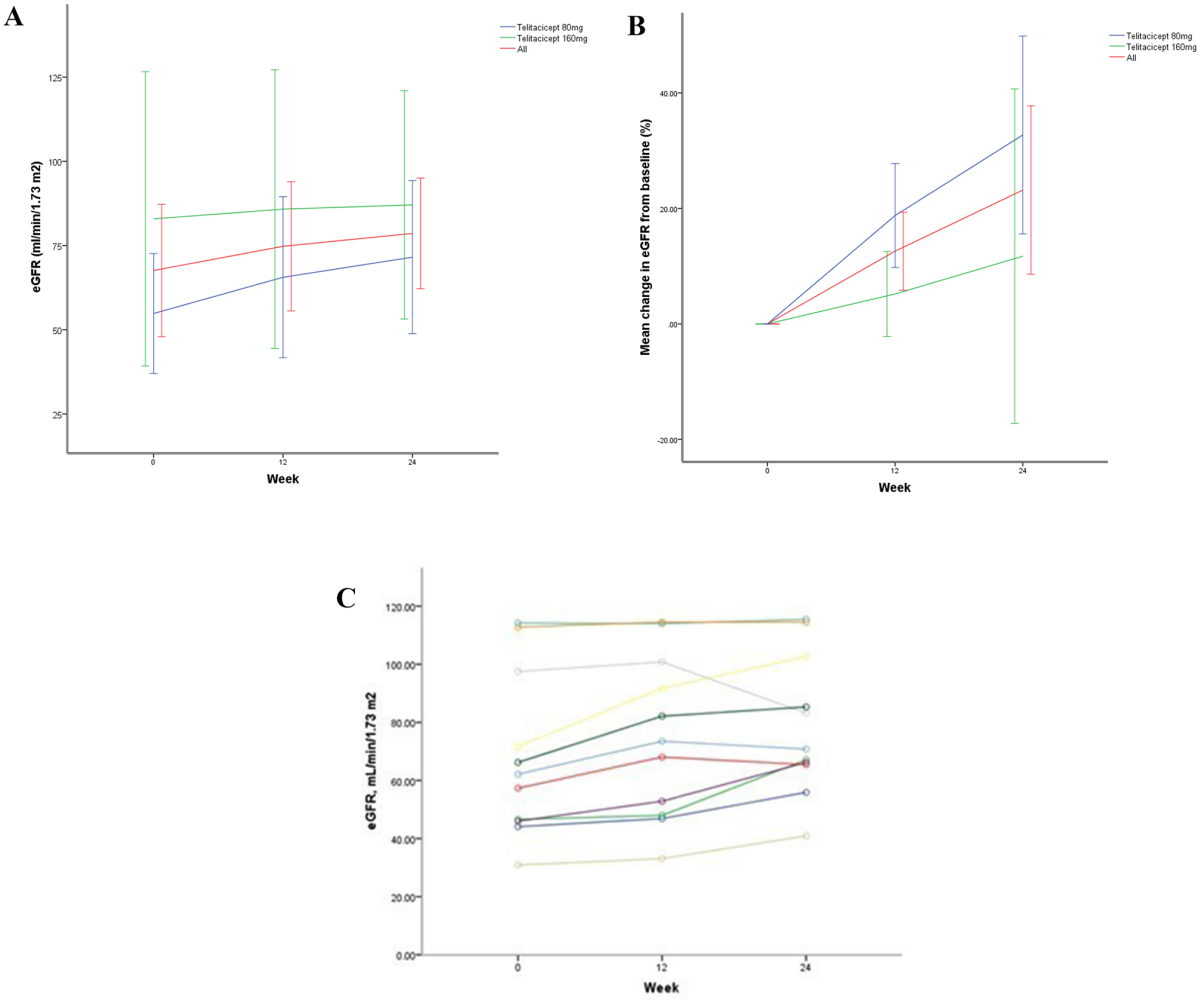
Changes in eGFR at baseline, 12 weeks and 24weeks. (**a**) Absolute mean changes in eGFR from baseline in patients receiving telitacicept (80 or 160 mg/week) at each visit. Data are expressed as mean values (bars indicate the standard error of the mean). (**b**) Mean change (%) in eGFR from baseline in patients receiving telitacicept (80 or 160 mg/week) at each visit. (**c**) Individual trajectories of eGFR for each patient in the cohort. eGFR indicates the estimated glomerular filtration rate calculated using CKD-EPI. CKD-EPI, chronic kidney disease epidemiology collaboration; eGFR, estimated glomerular filtration rate

The level of eGFR significantly improved from 67.59 ± 29.23 mL/min/1.73 m^2^ to 74.77 ± 28.56 mL/min/1.73 m^2^ at week 12, with mean absolute increase: 7.18 ± 6.54 mL/min/1.73 m² and mean percentage increase of 12.60% (95% CI: 5.84**–**19.36, *P* = 0.002), and further increased to 78.59 ± 24.46 mL/min/1.73 m^2^ at week 24, with mean absolute increase: 11.00 ± 12.16 mL/min/1.73 m² and mean percentage increase of 23.18% (95% CI: 8.60**–**37.76, *P* = 0.005). Similar to the trend observed in the full cohort, eGFR at 12 weeks in telitacicept 80 mg group increased from 54.82 ± 16.96 mL/min/1.73 m^2^ to 65.58 ± 22.78 mL/min/1.73 m^2^, with mean absolute increase: 10.76 ± 6.81 mL/min/1.73 m² and mean percentage increase of 18.78% (95% CI: 9.78**–**27.78, *p* = 0.003)), and 71.54 ± 21.66 mL/min/1.73 m^2^, with mean absolute increase: 16.72 ± 8.67 mL/min/1.73 m² and mean percentage increase of 32.72% (95% CI: 15.58**–**49.85, *p* = 0.004). In telitacicept 160 mg group, eGFR remained stable from 82.92 ± 35.19 mL/min/1.73 m^2^ to 85.81 ± 33.33 mL/min/1.73 m^2^ at week 12, with mean absolute increase: 2.89 ± 2.58 mL/min/1.73 m² and mean percentage increase of 5.19% (95% CI: − 2.17**–**12.55, *p* = 0.122), and 87.06 ± 27.30 mL/min/1.73 m^2^ at week 24, with mean absolute increase: 4.14 ± 12.95 mL/min/1.73 m² and mean percentage increase of 11.73% (95% CI: − 17.23**–**40.68, *p* = 0.324). (Figs. 2a, b). The individual trajectories of eGFR for all patients are provided in Figs. 2c.

Similarly, the sensitivity analysis (*n* = 9) confirmed the beneficial effect on renal function. The eGFR increased from 63.18 ± 28.05 mL/min/1.73 m² to 75.47 ± 23.84 mL/min/1.73 m² at week 24, with a mean percentage increase of 26.61% (95% CI: 9.32 to 43.90, *p* = 0.008).

### Secondary outcomes

#### Effects on hematuria

**Fig. 3:**
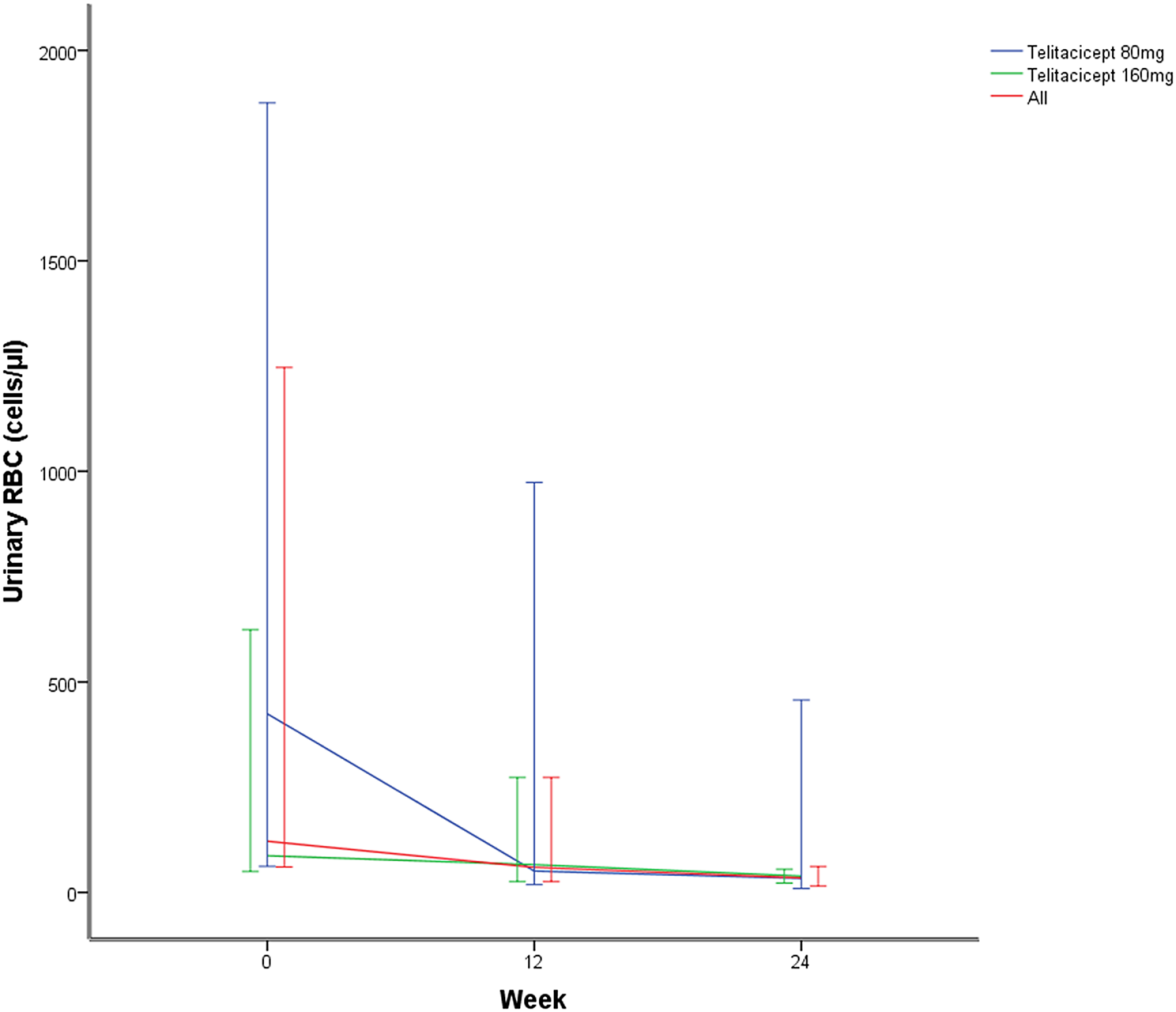
Median and IQR of urinary RBC counts (cells/µl) at baseline, 12 weeks and 24 weeks

In the whole telitacicept group, the urinary red blood cell (RBC) counts at 12 weeks decreased from 121.60 cells/µl (IQR 62.00–641.30) to 59.60 cells/µl (IQR 26.20–85.80) (*P* = 0.004), to 34.60 cells/µl (IQR 22.20–55.10) at week 24(*P* = 0.003). In telitacicept 80 mg group, from 424.55 cells/µl (IQR 70.10–1403.60) to 50.85 cells/µl (IQR 26.20–307.13) at week 12 (*P* = 0.028), to 33.30 cells/µl (IQR 13.53–160.63) at week 24 (*P* = 0.028). In telitacicept 160 mg group, from 87.20 cells/µl (IQR 54.90–372.95) to 66.10 cells/µl (IQR 26.15–179.55) at week 12 (*P* = 0.080), to 38.60 cells/µl (IQR 23.70–49.15) at week 24 (*P* = 0.043). (Figs. 3).

### Subgroup analyses

#### Comparison between 80 mg and 160 mg dose groups

No statistically significant differences were observed between the telitacicept 80 mg and 160 mg groups in the reduction of proteinuria (68.67% vs. 59.24%, *p* = 0.329), improvement in eGFR (32.72% vs. 11.73%, *p* = 0.126), or resolution of hematuria (81.92% vs. 62.39%, *p* = 0.177) at week 24.

### Impact of concomitant immunosuppression

The efficacy of telitacicept was comparable between patients receiving concomitant immunosuppressants (glucocorticoids ± other agents, *n* = 4) and those on telitacicept monotherapy (*n* = 7). The mean reduction in proteinuria at week 24 was similar between the combination therapy group and the monotherapy group (72.65% vs. 59.67%, *p* = 0.230). Likewise, no significant differences were found in eGFR improvement (21.53% vs. 24.12%, *p* = 0.927) or reduction in urinary RBC counts (78.82% vs. 69.74%, *p* = 0.788) between the two subgroups.

### Steroid-sparing effect

Telitacicept demonstrated a notable steroid-sparing effect. In our study, among the 4 patients using glucocorticoids, the mean dose decreased from 18.75 mg/d at baseline to 5.0 mg/d at the end of follow-up, representing 73.33% reduction in steroid dosage. In addition, 2 patients achieved complete steroid discontinuation.

### Treatment efficacy

At the last follow-up of each patient, the whole telitacicept group had 7 patients who had achieved PR, 3 patients who had achieved CR and an overall efficacy rate of 90.91%. In the 80 mg telitacicept subgroup there were 3 patients who had achieved PR, 3 patients who had achieved CR and an overall efficacy rate of 100%. Similarly, in the 160 mg telitacicept group, there were 4 patients who had achieved PR, 0 patients who had achieved CR and an overall efficacy rate of 80% (Table 2).

**Table 2:** Efficacy rates of participants during each follow-up period

Group	Overall efficacy rate, *n* (%)	CR, *n* (%)	PR, *n* (%)	Ineffective, *n* (%)
All telitacicept (*n* = 11)	10 (90.91)	3 (27.27)	7 (63.64)	1 (9.09)
Telitacicept 160 mg (*n* = 5)	4 (80)	0 (0)	4 (80)	1 (20)
Telitacicept 80 mg (*n* = 6)	6 (100)	3 (50)	3 (50)	0 (0)

CR, complete remission; PR, partial remission

### Safety and AEs

During the follow-up periods, no SAEs were observed. In the whole telitacicept group, only 2 patients in 160 mg telitacicept subgroup experienced local skin reactions (incidence of AEs was 18.18%).

## Discussion

IgA nephropathy, classified as an autoimmune-related kidney disease, is characterized primarily by the deposition of galactose-deficient IgA within the glomerular mesangial area [5]. Conventional treatment strategies for IgA nephropathy are primarily based on supportive care, often combined with immunosuppressive therapy [13]. Studies have shown that the B-cell stimulatory factors B lymphocyte stimulator (BLyS/BAFF) and A Proliferation-Inducing Ligand (APRIL) can accelerate the progression of IgA nephropathy [6–8].

Telitacicept, a dual-target inhibitor blocking both APRIL and BLyS, also impacts the secretion of autoantibodies by plasma cells [14]. This mechanism provides a rationale for its use in treating IgA nephropathy. The phase II telitacicept trial (NCT04291781; registered on February 28, 2020) have affirmed telitacicept’s efficacy and safety in IgA treatment [10], while further research must delineate its practical benefits in routine clinical applications.

Pioneering the real-world assessment of low-to-medium dose telitacicept (80–160 mg/week), this study reveals its efficacy in high-risk progressive IgAN adults refractory to conventional therapy. We observed a triad of renoprotective effects: a marked reduction in proteinuria (64.4%, *P* < 0.001), an improvement in estimated glomerular filtration rate (+ 23.2%, *P* = 0.005), and resolution of hematuria (71.5% RBC reduction), with a favorable safety profile.

To specifically address the potential confounding effect of concomitant immunosuppression, particularly from the two patients receiving potent agents (tacrolimus or mycophenolate mofetil), additional analyses were performed. A sensitivity analysis confirmed that after excluding these two patients, the efficacy in the remaining nine patients remained highly significant (61.24% proteinuria reduction, *p* < 0.001; 26.61% eGFR increase, *p* = 0.008). Furthermore, a pre-specified subgroup analysis comparing all patients on concomitant immunosuppressants (*n* = 4) versus those on telitacicept monotherapy (*n* = 7) revealed no statistically significant difference in efficacy (e.g., proteinuria reduction: 72.65% vs. 59.67%, *p* = 0.230). Together, these analyses strengthen the conclusion that the observed clinical benefits are primarily driven by telitacicept itself, rather than being solely attributable to concomitant immunosuppression.

Notably, the 80 mg dose regimen achieved comparable efficacy to 160 mg in both proteinuria reduction (68.67% vs. 59.23%) and eGFR improvement (+ 32.72% vs. + 11.73%), mirroring dose-response patterns observed in lupus nephritis trials [15]. A comparison of baseline characteristics between the dose groups was conducted to assess potential confounding. Overall, there were no statistically significant differences observed between the telitacicept 80 mg and 160 mg groups. A numerical disparity was noted in the median time from diagnosis to treatment (23 vs. 7 months, *p* = 0.051), a trend that warrants attention although it did not reach conventional statistical significance. The variance in treatment timing may reflect evolving real-world prescribing patterns and warrants exploration in larger cohorts.

This finding on lower-dose efficacy is of pivotal clinical importance when viewed in the context of accumulating real-world evidence. The compelling efficacy observed in our cohort is highly consistent with a recent study by Liu et al. in 16 pediatric patients (including 11 with IgAN), which reported a 79.12% reduction in 24-hour proteinuria at week 48 and a 62.5% complete remission rate [16]. This convergence of findings across independent centers and age groups strengthens the validity of our results despite the sample size constraint. Furthermore, while another large real-world study in adults by Weng et al. corroborated the proteinuria-reducing effect of telitacicept, it utilized the conventional 240 mg dose [17]. The comparable efficacy between 80 mg and 160 mg in our study aligns with a dose-response meta-analysis in systemic lupus erythematosus, which confirmed the efficacy of all doses (80 mg, 160 mg, 240 mg) but highlighted the need to define optimal dosing strategies that balance benefit and risk [15]. Thus, our results provide a rationale for considering a lower starting dose (e.g., 80 mg) and adjusting it based on individual therapeutic response and tolerability.

This study establishes that telitacicept provides rapid onset renoprotection in high-risk IgAN patients, with clinically significant proteinuria reduction and eGFR improvement emerging as early as Week 12. This early response offers a valuable therapeutic window for patients refractory to conventional therapies, enabling timely intervention before irreversible fibrosis develops. By Week 24, these benefits intensified, which demonstrated not only sustained efficacy but also progressive renal function recovery, thereby supporting telitacicept’s transition from short-term induction to long-term management in real-world practice.

Another clinically significant observation was telitacicept’s pronounced effect on hematuria, with urinary red blood cells decreasing by 71.54% at Week 24. This finding addresses an important therapeutic gap, as persistent hematuria independently predicts renal function decline in IgA nephropathy according to contemporary studies [18–20], yet remains unaddressed in current management guidelines. Wang et al. [21] reported median RBC decrease from 35.40 to 13.60 cells/µL (61.58% decline) consistent directionally with our study, supporting mechanistic coherence. Confirmatory trials are warranted to establish hematuria as a validated response marker.

Furthermore, telitacicept exhibited significant steroid-sparing effects: glucocorticoid doses decreased by 73.33% overall, with 18.18% of patients achieving complete discontinuation. Crucially, even in the 4 patients receiving concomitant immunosuppressants, no additive immunosuppressive risks were observed, underscoring its favorable safety profile during combination therapy. These findings provide critical evidence for optimizing IgAN treatment paradigms through targeted biologics.

While our therapeutic outcomes align with the established efficacy trajectory of the phase II telitacicept trial (NCT04291781), enhanced renoprotection was observed—characterized by superior proteinuria reduction (64.38% vs. 38–49%) and significant eGFR improvement (+ 23.18% vs. stabilization)—likely attributable to two pivotal factors: (1) the high prevalence of treatment-responsive pathology (72.73% exhibiting crescents with regenerative potential), and (2) concomitant immunosuppression in refractory cases (36.37% receiving glucocorticoids ± immunosuppressants, potentially amplifying efficacy though drug interactions require further elucidation).

The favorable safety profile observed in our cohort—characterized by only mild injection-site reactions (18.18%) and absence of serious infections—is consistent with the established mechanism of Telitacicept, which has fewer side effects than conventional medications as it is metabolized intracellularly without hepatic/renal clearance [14].

Study limitations include the small sample size, absence of a parallel control group, and relevant short observational time, which was only 24 weeks. Future investigations should validate these findings in large-scale and long-term trial.

In conclusion, low-to-medium dose telitacicept achieves rapid multisystem benefits in high-risk IgA nephropathy, contributing to reductions in proteinuria, increases in the eGFR, significantly reduces hematuria, and maintaining a favorable safety profile. The 80 mg regimen’s non-inferior efficacy underscores its viability for chronic management, addressing critical unmet needs.

## Data Availability

The data underlying this article will be shared on reasonable request to the corresponding author.
